# The Genetics of POAG in Black South Africans: A Candidate Gene Association Study

**DOI:** 10.1038/srep08378

**Published:** 2015-02-11

**Authors:** Susan E. I. Williams, Trevor R. Carmichael, R. Rand Allingham, Michael Hauser, Michele Ramsay

**Affiliations:** 1 Division of Ophthalmology, Department of Neurosciences, University of the Witwatersrand, Johannesburg, South Africa; 2 Duke Eye Center, Duke University, Durham, USA; 3 Duke Center for Human Genetics, Duke University, Durham, USA; 4 Division of Human Genetics, National Health Laboratory Service, School of Pathology, University of the Witwatersrand, Johannesburg, South Africa; 5 Sydney Brenner Institute of Molecular Bioscience, University of the Witwatersrand, Johannesburg, South Africa

## Abstract

Multiple loci have been associated with either primary open angle glaucoma (POAG) or heritable ocular quantitative traits associated with this condition. This study examined the association of these loci with POAG, with central corneal thickness (CCT), vertical cup-to-disc ratio (VCDR) and with diabetes mellitus in a group of black South Africans (215 POAG cases and 214 controls). The population was homogeneous and distinct from other African and European populations. Single SNPs in the *MYOC, COL8A2, COL1A1* and *ZNF469* gene regions showed marginal associations with POAG. No association with POAG was identified with tagging SNPs in *TMCO1, CAV1/CAV2, CYP1B1, COL1A2, COL5A1, CDKN2B/CDKN2BAS-1, SIX1/SIX6* or the chromosome 2p16 regions and there were no associations with CCT or VCDR. However, SNP rs12522383 in *WDR36* was associated with diabetes mellitus (p = 0.00008). This first POAG genetic association study in black South Africans has therefore identified associations that require additional investigation in this and other populations to determine their significance. This highlights the need for larger studies in this population if we are to achieve the goal of facilitating early POAG detection and ultimately preventing irreversible blindness from this condition.

Glaucoma, a neurodegenerative condition characterized by progressive damage to the retinal ganglion cells and optic nerve fibers resulting in visual field loss, is the most important cause of irreversible visual loss in South Africa, as it is worldwide^1^,^2^,^3^. In South Africa, primary open angle glaucoma (POAG) is the commonest form of glaucoma and has a prevalence of 2.8% over the age of forty years^2^,^3^,^4^. This is about three times the prevalence in Caucasian populations and is an illustration of the recognized racial differences in POAG prevalence^5^,^6^,^7^. The racial differences, along with the strong familial association of POAG, form part of the evidence for a genetic basis for this disease^8^,^9^. Given that POAG is amenable to treatment when it is detected early, identifying genetic risk factors could potentially offer the prospect of early diagnosis. For example, treatment could be initiated in the presence of genetic risk factors in pre-perimetric glaucoma with ocular hypertension or in early glaucomatous optic neuropathy with a normal intra-ocular pressure (IOP) thus preventing visual loss. POAG is, however, phenotypically and genetically heterogeneous^10^,^11^.

Only a small proportion of POAG appears to be inherited as a monogenic trait that is autosomal dominant^12^,^13^. Family studies have successfully identified several loci including three genes that are associated with monogenic forms of POAG - *MYOC*^14^, *WDR36*^15^, and *OPTN*^16^,^17^. The majority of POAG, however, is thought to be of complex, multifactorial inheritance^13^. Association studies using common genetic variants are a powerful tool for uncovering significant genes in common conditions with multifactorial inheritance^18^,^19^,^20^. Genome-wide association (GWA) studies are limited in that they require very large sample numbers. They are expensive and they may not adequately cover all regions of the genome. In populations of African descent, where there is greater genetic variation, these limitations are aggravated^21^. Candidate gene association studies are a suitable alternative to identify the underlying genetic components of POAG^12^. Strong phenotypic data on the subjects strengthens the value of this type of study.

Candidate gene association studies have been used to identify genetic risk factors for POAG in several diverse populations, but never in black South Africans. The identification of disease-associated alleles in different populations is important to understanding their contribution to the pathogenesis of POAG. We therefore undertook this candidate gene association study in black South Africans. In selecting the candidate regions, we chose known glaucoma genes (*MYOC*, *WDR36* and *CYP1B1*^22^), regions that have been identified in POAG association studies in other populations (the locus at chromosome 2p16^23^, *CAV1/CAV2*^24^, *CDKN2B/CDKN2B-AS1*^25^,^26^,^27^, *TMCO1*^25^ and *SIX1/SIX6*^27^,^28^), candidate genes for central corneal thickness (CCT) (the collagen genes *COL1A1*^29^, *COL1A2*^29^, *COL5A1*^30^,^31^ and *COL8A2*^32^ and the region of *ZNF469*^30^,^31^) and candidate genes for optic nerve parameters (*CDKN2B*^33^, *SIX1/SIX6*^33^ and *ATOH7*^33^).

The purpose of this study was to evaluate the association between POAG in black South Africans and single nucleotide polymorphisms (SNPs) in these genomic regions using a haplotype-tagging approach. Secondary aims were to evaluate the association in this population with CCT and *COL1A1, COL1A2, COL5A1, COL8A2* and *ZNF469*; vertical cup-to-disc ratio (VCDR) and *CDKN2B, SIX1/6* and *ATOH7*; and diabetes mellitus with all the candidate genes.

## Methods

### Population and sample

The study adhered to the tenets of the Declaration of Helsinki. Written informed consent was obtained from all participants. The research was reviewed and approved by the University of the Witwatersrand Human Research Ethics Committee (Ethics clearance: M10216) and the methods were carried out in accordance with the approved guidelines.

Participants were enrolled at the St John Eye Hospital in Soweto, Johannesburg, Gauteng Province, South Africa. The hospital is the eye department of Chris Hani Baragwanath Hospital, the largest hospital in South Africa. It is a teaching hospital for the University of the Witwatersrand and a public sector hospital managed by the Gauteng Department of Health. The hospital serves the greater Soweto region, but is a tertiary referral center, so it receives referrals from a larger area of Gauteng. Gauteng is the smallest of South Africa's provinces, but the most populous, with a population of 12.3 million as of the 2011 South African Population Census^34^. The population of Soweto is an urban population with representatives from all South African ethnic groups.

Unrelated self-identified black South African POAG patients and unrelated gender- and ethnicity-matched controls were invited to participate in the study. Admixed individuals in South Africa self-identify as ‘Coloured’ and only make up a small proportion of the population served by the hospital. They were not included in this study. The participants' ethnic ancestry was assigned by their mother tongue and that of their parents and grandparents. A medical history was recorded in all participants that included the length and type of treatment for common medical conditions such as hypertension and diabetes mellitus. The latter was further grouped into Type I or Type 2 diabetes mellitus (T2DM). Each participant underwent a comprehensive ophthalmological examination. VCDR was determined by dilated fundoscopy of the optic nerve head using a superfield lens. Intra-ocular pressure (IOP) was measured using a slit-lamp mounted Goldmann applanation tonometer. The IOP at diagnosis was ascertained from the hospital records. CCT was measured with the Reichert IOPac handheld pachymeter. The visual field assessment was performed on an Oculus automated perimeter. A structural assessment was performed with scanning laser polarimetry using the GDxVCC™. POAG was defined by the presence of typical glaucomatous optic neuropathy (VCDR greater than 0.7 or a focal notch or both) with corresponding visual field loss, open drainage angles on gonioscopy and absence of a secondary cause for glaucomatous optic neuropathy. The control participants were in an older age group (all controls were 50 years or older) to increase the diagnostic certainty of the ‘control’ label and had no evidence of exfoliation syndrome or glaucoma and no familial history of glaucoma.

### DNA extraction, SNP selection and genotyping

Genomic DNA was extracted from peripheral blood using a salting out procedure modified from Miller et al.^35^ The DNA from each individual in the study population was normalized to a concentration of 50 ng/μl. The DNA samples were stored in the sample repository of the National Health Laboratory Services (NHLS) Molecular Genetics Laboratory in the Division of Human Genetics of the University of the Witwatersrand (Johannesburg). Aliquots (200 μl) of the DNA were couriered (on ice) to the Duke Center for Human Genetics (Durham, NC) and were stored in that Biobank.

Genotyping was first performed at the University of the Witwatersrand on the medium-throughput Illumina BeadXpress platform using the Illumina Goldengate Assay that targets specific SNPs in genomic DNA samples (Illumina, San Diego, CA). Thirteen candidate genomic regions were investigated in this manner, namely *MYOC, CDKN2B, CAV1/CAV2, CYP1B1, WDR36, COL1A1, COL1A2, COL5A1, COL8A2, ZNF469, SIX1/SIX6, ATOH7* and the chromosome 2p16 locus. The candidate region selection took place from January to June 2011. The selection was based on a literature review at that time^11^,^12^,^13^,^22^,^23^,^24^,^29^,^30^,^31^,^32^,^33^. There are many other genes/loci that have been shown to be associated with POAG that were not, therefore, included.

SNPs within these regions that had shown an association in other studies and in other populations were included in the assay design. We also included SNPs in the *MYOC* gene that we had identified in a separate study by sequencing the exons in a subgroup of these patients^36^. Tagging SNPs were designed using genotype data from the HapMap project (www.hapmap.org - HapMap data release #27) in African (YRI – Yoruba from Nigeria) samples using the ‘Tagger Multimarker’ protocol which aggressively searches for multi-marker predictors to capture all alleles of interest (SNPs and/or haplotypes)^37^. We used a minimal coefficient of determination (r^2^) at which alleles were to be captured of 0.8 and we only selected SNPs with a minor allele frequency of more than 0.05. We compared the YRI HapMap tagger data with that for the Luhya in Webuye, Kenya (LWK) and the Maasai in Kinyawa, Kenya (MKK) using Tagger in Haploview (version 4.2) and added SNPs to capture further genetic diversity. The chromosome 2p16 region and *COL5A1* could not be adequately covered with tag SNPs, therefore only the previously associated SNPs were used in these regions.

Lao *et al*.^38^ identified ten SNPs that were informative of geographic population structure and genetic ancestry. The ten SNPs were included in the assay design together with eight SNPs that discriminate among southern African populations and that were identified locally (unpublished data)^39^. These eighteen SNP markers were therefore considered informative of ancestry and were included in the assay in addition to the candidate region SNPs for the population substructure analysis.

The GoldenGate assay was performed according to the manufacturer's instructions (Illumina, San Diego, CA). Genotyping results were derived from the BeadXpress fluorescence data with the BeadStudio software package (Illumina, San Diego, CA). The software was also used for quality control on samples and SNPs. Samples were excluded if there was evidence of contamination, call rates less than 0.95, problems with allele specific extension, PCR uniformity or hybridization or inconsistency of gender estimation (from the genotyping) and known gender. SNPs were excluded if there was low call frequency, poor cluster separation, questionable clustering or a low ‘50% GC’ rate (GC is an estimation of the confidence of the genotype call for any given sample with a value between 0 and 1 and the ‘50% GC’ rate is the median GC for all samples at one SNP).

SNPs from *TMCO1, CAV1/CAV2, CDKN2B-AS1* and *SIX1/SIX6* were then genotyped using TaqMan allelic discrimination assays at Duke University. These SNPs were selected because they have been evaluated in two other populations of African Ancestry^40^. Assay-On-Demand products were used with the ViiA7 Realtime PCR system with 384-well block according to the standard protocols of the manufacturer (Applied Biosystems, Foster City, CA). The fluorescent signal intensity was measured directly in the reaction well by the ABI ViiA7 Sequence Detector (Applied Biosystems). For quality control purposes samples from two individuals were duplicated across all plates and two CEPH (the Centre d'Etude du Polymorphisme Humain, Foundation Jean Dausset, Paris, France) standards were included in each 96- well plate. Genotyping efficiency of at least 95% and matching genotypes of quality-control samples within and across all plates was required for inclusion in the statistical analysis.

### Statistical analysis

*Power calculations* were performed using Quanto Version 1.2. A ‘log-additive’ mode of inheritance was assumed. The Odds Ratio (OR) that could be detected 80% of the time with 95% confidence in an unmatched case-control study of genetic association in POAG in South Africa (assuming a disease prevalence of 2.8%) in 215 cases and controls was calculated for population disease allele frequencies ranging from 0.01 to 0.99. This study is adequately powered to detect odds ratios of 0.6 or less and 1.5 or more when the frequencies of the disease alleles in the population are between 0.2 and 0.7. Where the population disease allele frequencies are greater or less than this, this sample size is only powered to detect more significant odds ratios.

*Descriptive statistical analyses* were performed using STATA version 12. To describe the demographic characteristics, we used mean ± standard deviation (SD) for continuous variables, and we summarized categorical variables by percentages. For comparisons of continuous variables, we used the t-test with p values that were two-tailed with significance set at p < 0.05. For comparisons of categorical variables we used Fisher's exact test or χ^2^ analysis. P values < 0.05 were considered to be significant. For the analysis of continuous traits present in both eyes (CCT and VCDR), we used the average value of the two eyes or the value of only one eye where the fellow eye could not be measured or, in POAG participants, if the fellow eye was untreated (in unilateral glaucoma or blindness). To adjust for the confounding variables of age and gender we used a logistic regression model with age and gender as covariates. An adjusted p value was considered significant if p < 0.05.

*Association analyses* were performed using PLINK (v1.07). The minimum genotyping rate for SNPs and individuals to be included in the statistical analyses was set to be 90%. Hardy-Weinberg equilibrium was assessed by using the χ^2^ test. SNPs that were not in Hardy-Weinberg equilibrium in the group of controls were excluded from further analysis. Genotype frequencies of POAG cases and controls were compared by logistic regression with adjustment for age and gender. Association of the allele frequencies of SNPs in *COL1A1, COL1A2, COL5A1*, *COL8A2* and *ZNF469* with CCT and the allele frequencies of SNPs in *CDKN2B, SIX1* and *SIX6* (only the SNPs genotyped with the BeadXpress platform), and *ATOH7* with VCDR was evaluated by using linear regression after adjusting for diagnosis (POAG or control), age and gender in the study population. Genotype frequencies of diabetic and non-diabetic participants were compared by logistic regression with adjustment for diagnosis (POAG or control), age and gender.

Multiple comparisons of SNPs covering each investigated genomic region were corrected using the Bonferroni method.

*Structure analysis* was performed using EIGENSOFT (v 4.2). A principal components analysis on the data was used to identify structure and correlation between cases/controls and eigenvectors of the structure. Comparisons of this dataset with other African and European datasets were performed to detect admixture and were plotted using R (v 2.15.2).

## Results

BeadXpress SNP genotyping was successful in 429 participants (215 POAG patients and 214 controls). Demographic and clinical features of the study subjects are summarized in Table 1. The participants were all self-identified black South Africans. The majority spoke Southern Bantu languages. The POAG and control groups were alike in the proportions speaking the different languages and this reflected the data from the South African census in 2011 (supplementary figure)^34^.

BeadXpress genotyping was performed on 171 SNPs that were selected to tag the common genetic variations in *MYOC, CDKN2B, CAV1/2, CYP1B1, WDR36, COL1A1, COL1A2, COL5A1, COL8A2, ZNF469, SIX1/6, ATOH7* and the chromosome 2p16 locus. Seven SNPs failed quality control testing using BeadStudio software and were excluded from further analysis. A further five SNPs were excluded because the genotype distributions in the control group were not consistent with Hardy-Weinberg equilibrium (p < 0.01). The analyses were therefore performed on a total of 159 SNPs from these regions. A further 18 SNPs from across the genome that were considered to be informative of ancestry were included in the genotyping for use only in the structure analysis.

**Figure 1:**
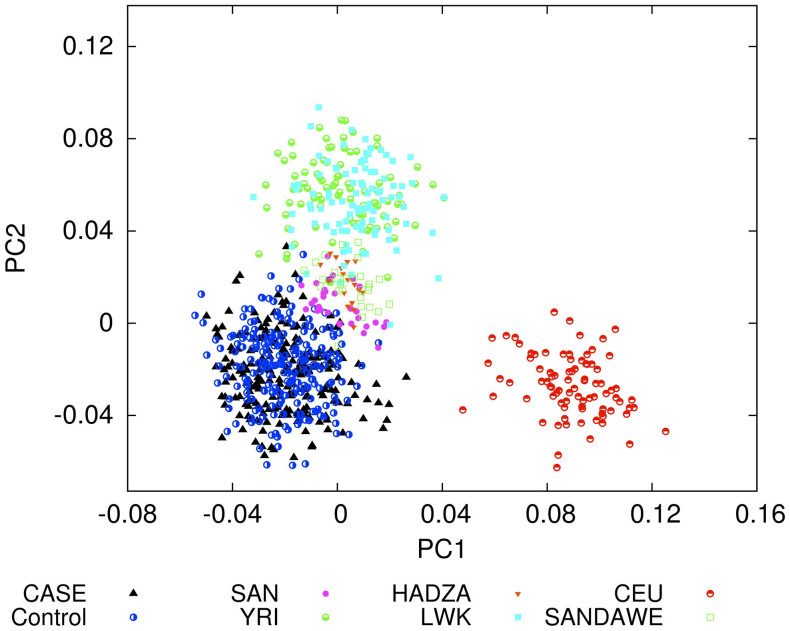
Structure analysis of 137 SNPs in common in this data (CASE, POAG; Control, Controls) and in other African populations (SAN, San from Southern Africa; HADZA, Hadzabe from Tanzania; YRI, Yoruba from Nigeria; LWK, Luhya from Kenya; SANDAWE, Sandawe from Tanzania) and a European population (CEU, Utah residents with European ancestry).

A principal component analysis of 137 of the genotyped SNPs (including the 18 SNPs informative of ancestry) that were common to this and HapMap African (YRI, LWK) and European (CEU) and other African (Khomani Bushmen, Hadza and Sandawe)^41^ datasets showed little structure in the study group and no difference between POAG or control status and eigenvectors of the structure. Combining the data with these other African and European data sets revealed a homogeneous population distinct from the other populations with no evidence of admixture (Figure 1).

TaqMan allelic discrimination assays were performed on 49 SNPs from *TMCO1, CAV1/CAV2, CDKN2B-AS1* and *SIX1/SIX6*. This was successful in a subgroup (179 POAG participants and 187 controls) of the participants in whom BeadXpress genotyping was successful. No SNPs failed quality controls. A SNP in the *CAV1* gene (rs3779512) had a genotype distribution in the control group that was not consistent with Hardy-Weinberg equilibrium (p < 0.01) and was excluded from further analysis. The panel of SNPs included 9 SNPs that were also genotyped with the BeadXpress platform. The genotyping results from the TaqMan assays and the BeadXpress genotyping for each individual for each of these SNPs were identical and therefore confirmed the validity of both methods. For these 9 SNPs only the BeadXpress results are reported.

The panel of SNPs genotyped in this case-control study therefore consisted of 198 SNPs. The chromosomal location and the number of SNPs genotyped and analyzed in each gene or region are shown in Table 2.

There were four SNPs with a nominally significant association with POAG in this population (p < 0.05). These were individual SNPs in *COL8A2* (rs6693322), *MYOC* (rs235917), *COL1A1* (rs16948744) and *ZNF469* (rs9925231) (Table 3 and Supplementary Table 1). The associations did not withstand correction for multiple testing.

There were 71 SNPs in the genomic regions thought to be associated with CCT, namely *COL1A1, COL1A2, COL5A1*, *COL8A2*, and the *ZNF469* region. In the linear regression association analysis between these SNPs and CCT, the C allele of SNP rs2521206 in *COL1A2* and the T allele of SNP rs7500824 in the *ZNF469* region were both weakly associated with a thinner central cornea in the study population after adjusting for age, gender and diagnosis (POAG or control) (Supplementary Table 2). This association did not withstand correction for multiple testing. We found no evidence of association between the 31 SNPs in the regions of *CDKN2B, SIX1*/SIX*6*, and *ATOH7* and VCDR in this population (Supplementary Table 3).

**Figure 2:**
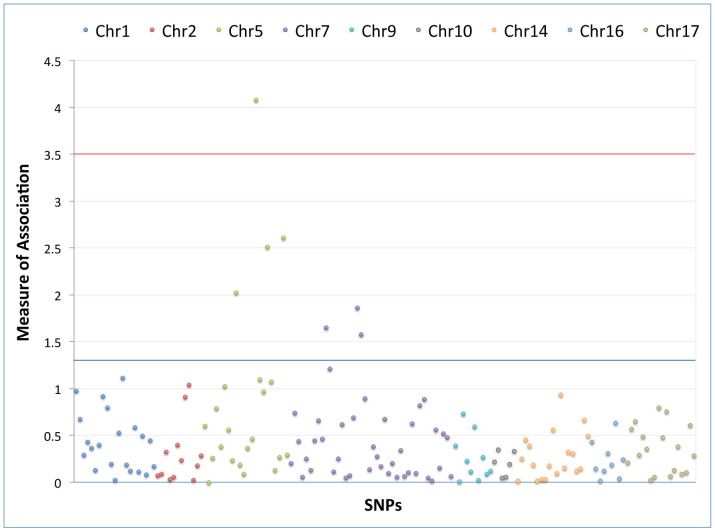
Summary of association of SNPs with diabetes mellitus by logistic regression. The measure of association is represented on the y-axis by -log_10_(p). The association was calculated by logistic regression adjusted for diagnosis (POAG or control), age and gender. The SNPs represented are in *COL8A2* (chromosome 1), *MYOC* (chromosome 1), *CYP1B1* (chromosome 2), Chromosome 2p16 region, *WDR36* (chromosome 5), *COL1A2* (chromosome 7), *CAV1/CAV2* (chromosome 7), *CDKN2B* (chromosome 9), *COL5A1* (chromosome 9), *ATOH7* (chromosome 10), *SIX1/SIX6* (chromosome 14), *ZNF469* (chromosome 16) and *COL1A1* (chromosome 17). The blue line represents p = 0.05. The red line represents the Bonferroni corrected P = 0.0003 (0.05/159).

Single SNP association testing with a diagnosis of diabetes mellitus in the study population was only performed on the 159 SNPs successfully genotyped with the BeadXpress platform. Regions with significant SNP associations (p < 0.05) after adjustment for age, gender and diagnosis (POAG or control) were identified within the *WDR36* and *COL1A2* genes (Figure 2).

The association of diabetes mellitus with SNP rs12522383 in *WDR36* remained significant after correction for multiple testing. A diabetic subgroup analysis of association with SNP rs12522383 confirmed the association for Type 2 diabetes mellitus (Table 4).

## Discussion

The study population of black South Africans was representative, from an ethnic affiliation perspective, of the region in which the study was conducted. It consisted of individuals with different ethnic and tribal affiliations evidenced by their different home languages. However the languages (except Afrikaans) are all Southern Bantu languages (part of the Niger-Kordofanian linguistic macrofamily) and a structure analysis confirmed that the population is homogeneous but distinct from other African populations with no evidence of admixture. A high proportion of the study population were known to be hypertensive and/or diabetic which is consistent with the age of the study group and with this population, where these are both significant health problems^42^. The control group was deliberately selected to be older to ensure better accuracy in determining control status, but as a result more of the controls were hypertensive. The controls were clinic patients in whom the most common diagnosis was cataracts followed by a routine diabetic eye assessment, therefore both selection bias and age were explanations for the higher prevalence of diabetes in the control group. Likewise, selection bias and better ophthalmological follow-up of diabetic patients that also have POAG may explain the greater proportion of control diabetics with diabetic retinopathy. The greater proportion of both diabetics and diabetic retinopathy in the control group is a potential confounder that was not included in the POAG association statistical model. The inclusion of age as a covariate in the regression models may have mitigated this somewhat. POAG diagnosis, on the other hand, was included as a covariate in the regression models for diabetes, CCT and VCDR.

The phenotypic characteristics of the POAG patients were similar to those in other populations from Sub-Saharan Africa, but differed from those in developed nations^43^,^44^,^45^,^46^,^47^,^48^. The POAG participants had advanced disease with more than half having end-stage disease and severe visual impairment as a result of glaucoma. More than half of the participants had undergone surgical glaucoma drainage procedures. The mean age at diagnosis was relatively young at 55 years, however age at diagnosis does not necessarily equate with age at onset. The age of onset could be many years before the age at diagnosis in the context of participants presenting, as they did in this study, with advanced disease. It is probable, therefore, that the proportion of patients with juvenile onset glaucoma was underestimated. The IOP at diagnosis was high with very few of the participants having normal tension glaucoma. Glaucoma is under-diagnosed in this population, which explains how few of the cohort had a family history of glaucoma^2^. However the contribution of genetics to the pathogenesis of the disease in this population is presumably important because significantly more of the POAG group than the control group had a family history of blindness.

This study did not identify a significant association with POAG in black South Africans and the seven SNPs genotyped in the chromosome 2p16 region that had all shown significant associations in the Afro-Caribbean population of Barbados^23^. The study was well powered to detect the magnitude of risk reported in that study. The minor allele frequencies of the SNPs in this study were different to the original study, suggesting that despite the commonality of African descent, these two populations are not similar. As with our study, no association was shown with this region and POAG in Japanese and Korean cohorts^49^,^50^. Only a weak association was found with POAG and one SNP in this region in African-Americans (but not in Ghanaians)^51^. This suggests that the reported association may be specific to the original population in which it was identified. It may represent genetic drift or region specific selection pressures. Alternately there may be an unidentified causal variant in this region with different levels of linkage disequilibrium with the genotyped SNPs in different populations.

No significant associations with POAG were identified with SNPs in known glaucoma genes (*MYOC, WDR36* and *CYP1B1*), in regions that have been identified in association studies in other populations (*TMCO1*, *CAV1/CAV2, CDKN2B* and *SIX1/SIX6*) and other candidate genes for heritable ocular quantitative traits associated with POAG (the collagen genes *COL1A1, COL1A2* and *COL5A1, COL8A2*, the region of *ZNF469*, *CDKN2B, SIX1/SIX6* and *ATOH7*). However, this study was powered to detect moderate genetic risk assuming allele frequencies in the range of 0.2 to 0.7. The allele frequencies in this study were frequently lower than those reported in the literature. Smaller genetic effects cannot be excluded. Different genetic structures among different populations may be responsible for some of the conflicting results reported in the literature^52^. Liu et al.^40^, in their findings from a much larger association study of glaucoma in populations of African ancestry, concluded that genetic associations for POAG found in Caucasian populations play a smaller role in African POAG. Because of our small sample size we were underpowered to replicate any of the findings in that study.

This study was further limited by the selection of candidate genes and the selection of SNPs within each locus. Many other genes/loci have been shown to be associated with POAG, and their association in this population was not evaluated. The selection of tagging SNPs used linkage disequilibrium from HapMap data from the Yoruba in Ibadan, Nigeria (YRI), the Luhya in Webuye, Kenya (LWK) and the Maasai in Kinyawa, Kenya (MKK). However, the structure analysis revealed that this South African population was distinct from the YRI and LWK. Exploring linkage disequilibrium within a Southern African population might have yielded different SNPs.

Diabetes mellitus is heritable and considered to have an association with POAG, therefore it was included in this genetic association study related to POAG. The association of diabetes mellitus with POAG is, however a controversial one with conflicting results from population-based studies^53^,^54^,^55^,^56^ and epidemiological studies^57^,^58^. There is however an overlap in the risk factors for developing both diabetes and POAG and they are both diseases where vascular components contribute to their pathophysiology^59^. There is a need for further research to clarify the relationship between these two diseases and this should include genetic studies. In this study, a significant association with a SNP in *WDR36* (rs12522383) that withstood correction for multiple testing was identified with a self-reported history of diabetes mellitus in this population, and more specifically, T2DM. This SNP and three others that showed more marginal associations with T2DM were not associated with POAG. The genetic association of *WDR36* and diabetes mellitus is not among the 3806 genes reported with diabetes mellitus and recorded in the Phenopedia section of the Human Genome Epidemiology Network (HuGENet™) as of February 2014^60^. A weakness of this finding is that body mass index was not recorded in this study, so the association results were not corrected for body mass index. The association with diabetes mellitus we have detected needs to be replicated in order for its significance to be confirmed. Confirmation of an association with diabetes and a recognized POAG locus would strengthen the evidence for an independent association between these two disease entities.

This study has identified an association that requires additional investigation in this and other populations to determine its significance: that of *WDR36* with T2DM. Furthermore the results add to the evidence that the POAG genetic susceptibility alleles found in other populations play a reduced role in populations of African ancestry underscoring the need for large genome-wide association studies in these populations.

## Author Contributions

This is a sub-study toward SEIW's PhD. S.E.I.W., M.R. and T.R.C. conceived and designed the study. S.E.I.W. performed the data analysis and interpretation. S.E.I.W. wrote the main manuscript text. T.R.C., R.R.A., M.H. and M.R. reviewed the manuscript.

## Supplementary Material

Supplementary Information Supplementary figures and tables

## Figures and Tables

**Table 1:** Demographic and clinical features of study subjects in whom BeadXpress genotyping was successful

Group		Total	POAG	Control	p	Adjusted p
n		429	215	214		
Female (%)		222 (51.8)	107 (49.8)	115 (53.7)	0.411‡	
Age (years)	Range		22–87	50–91		
	Mean ± SD		59.8 ± 13.4	70.2 ± 8.3	**<0.001**§	
FAMILY HISTORY						
Family history of glaucoma (%)			15 (7.0)	N/A		
Family history of blindness (%)			52 (24.2)	15 (7.01)	**<0.001**‡	
HOME LANGUAGE					0.492¶	
	Afrikaans (%)	8 (1.9)	4 (1.9)	4 (1.87)		
	IsiNdebele (%)	5 (1.2)	1 (0.5)	4 (1.87)		
	Sepedi (%)	33 (7.7)	16 (7.4)	17 (7.94)		
	Sesotho (%)	70 (16.3)	32 (14.9)	38 (17.76)		
	Siswati (%)	5 (1.2)	1 (0.5)	4 (1.87)		
	Xitsonga (%)	23 (5.4)	12 (5.6)	11 (5.14)		
	Setswana (%)	69 (16.1)	30 (14.0)	39 (18.22)		
	Tshivenda (%)	11 (2.6)	8 (3.7)	3 (1.40)		
	IsiXhosa (%)	36 (8.4)	21 (9.8)	15 (7.01)		
	IsiZulu (%)	167 (38.9)	89 (41.4)	78 (36.45)		
	Other (%)	2 (0.5)	1 (0.5)	1 (0.47)		
MEDICAL HISTORY						
DM (%)		129 (30.1)	49 (22.8)	80 (37.38)	**0.001**‡	**0.037**
	Type I	7 (5.4)	5 (10.2)	2 (2.50)	0.104¶	0.211
	Diabetic retinopathy	30 (23.3)	3 (6.1)	27 (33.75)	**<0.001**¶	**<0.001**
HT (%)		266 (62.0)	118 (54.9)	148 (69.17)	**0.002**‡	0.81
Severe visual impairment (%)			114 (53.0)			
Glaucoma drainage surgery (%)			119 (55.4)			
Age at diagnosis (years)	Range		17–84			
	Mean ± SD		54.5 ± 13.8			
	Juvenile onset (%)		25 (11.6)			
IOP	Range		18–68	6–19		
	Mean ± SD		35.2 ± 9.5	13.4 ± 2.7	**<0.001**§	
	NTG (%)		6 (3.4)			
CCT	Range		379–586	420–609		
	Mean ± SD		506.0 ± 38.4	513.8 ± 37.2	**0.049**§	**0.004**
VCDR	Range		0.3–1.0	0.1–0.7		
	Mean ± SD		0.90 ± 0.13	0.37 ± 0.13	**<0.001**§	

DM, diabetic; HT, hypertensive; Severe visual impairment, Snellen visual acuity <20/200 in at least one eye secondary to glaucoma; Juvenile onset, POAG diagnosis before the age of 35 years; IOP, IOP at diagnosis for POAG subjects or at enrolment for control subjects; NTG, normal tension glaucoma (IOP < 20 mmHg); CCT, central corneal thickness; VCDR, vertical cup-to-disc ratio; N/A, not applicable (an exclusion criterion for participation).

Adjusted P, p adjusted for age and gender in a logistic regression model.

^‡^Pearson's χ^2^ test.

^¶^Fisher's exact test.

^§^T-test.

**Table 2:** The number of SNPs successfully genotyped in each genomic region

Genomic region	Chromosomal Location	Number of SNPs
TMCO1*	chr1: 163951690..164006222	8
*MYOC*	chr1: 169871181..169888396	18
Chromosome 2p16	chr2: 51000000..52000000	7
*CYP1B1*	chr2: 38148250..38156796	5
CAV1/CAV2**	chr7: 115926680..115988466	20
*COL8A2*	chr1: 36333433..36338437	3
*COL1A2*	chr7: 93861809..93902000	29
*COL1A1*	chr17: 45600000..45633999	18
*COL5A1*	chr9: 136673473..136876507	4
*CDKN2B*	chr9: 21992906..21999312	7
CDKN2BAS-1*	chr9: 21994791..22121097	17
*ATOH7*	chr10: 69660000..69680000	6
SIX1/SIX6**	chr14: 60045775..60185934	25
*WDR36*	chr5: 110455769..110494099	22
*ZNF469*	chr16: 86855000..86900000	9

The genotyping was performed using the BeadXpress platform except where * denotes TaqMan genotyping and ** denotes both BeadXpress and TaqMan genotyping.

**Table 3:** Associated SNPs (p < 0.05) in the genetic association of single SNPs with POAG in the study population using logistic regression modeling with the justification of age and gender. None withstood Bonferroni correction for multiple comparisons (p < 0.00025)

Genomic region	SNP	MA	MAF POAG	MAF Controls	p	OR (95% CI)
*COL8A2*	rs6693322	G	0.107	0.070	0.0139	1.92 (1.14–3.24)
*MYOC*	rs235917	A	0.084	0.040	0.0140	2.32 (1.19–4.52)
*COL1A1*	rs16948744	G	0.370	0.437	0.0143	0.68 (0.5–0.93)
*ZNF469*	rs9925231	A	0.502	0.428	0.0225	1.43 (1.05–1.93)

MA, minor allele; MAF, minor allele frequency; OR (95% CI), Odds ratio (95% confidence intervals).

**Table 4:** Diabetic subgroup analysis for association with rs12522383 (T allele) in *WDR36* using logistic regression modeling with the justification of diagnosis (POAG or control), age and gender

Diabetic phenotype	n cases/controls	MAF cases:controls	p	OR (95% CI)
**All diabetics**	**129/300**	**0.256:0.143**	**0.00008**	**2.06 (1.43–2.95)**
Type 1	7/422	0.357:0.174	0.07713	
**Type 2**	**122/307**	**0.250:0.148**	**0.00043**	
Diabetic retinopathy	30/399	0.233:0.173	0.26460	

OR (95% CI), Odds ratio (95% confidence intervals).

MAF, minor allele frequency.

Bold indicates significance.
