# Supplementary material for: The Genetics of POAG in Black South Africans: A Candidate Gene Association Study
Source: Sci Rep. 2015 Feb 11;5:8378. doi: 10.1038/srep08378 (PMC4323640; doi:10.1038/srep08378)
Supplement: Supplementary Information — Supplementary figures and tables [file srep08378-s1.doc]

The Genetics of POAG in Black South Africans: A Candidate Gene Association Study

Susan E I Williams, Trevor R Carmichael, R Rand Allingham, Michael Hauser, Michele Ramsay

**SUPPLEMENTARY MATERIAL**

Supplementary Figure:

**First language spoken:** Study population compared with South African Census 2011 results in self-identified black Africans. The languages are presented along the x-axis. The y-axis represents the frequency in percent.

C2011-RSA, census 2011 in black Africans in the whole country

C2011-Gauteng, census 2011 in black Africans in Gauteng

C2011-Johannesburg, census 2011 in black Africans in Johannesburg

Supplementary Table 1

| Supplementary Table 1. The genetic association of single SNPs with POAG in the study population using logistic regression modeling with the justification of age and gender | | | | | | | | | | |
| --- | --- | --- | --- | --- | --- | --- | --- | --- | --- | --- |
| **Genomic** | **SNP** | **MA** | | | **MAF** | | **MAF** | | **p** | **OR (95% CI)** |
| **region** |  |  | | | **POAG** | | **Controls** | |  |  |
| *TMCO1*** | rs10800149 | A | | | 0,19 | | 0,19 | | 0.5628 | 0.89 (0.6 - 1.32) |
| *TMCO1*** | rs10800150 | C | | | 0,49 | | 0,44 | | 0.2212 | 1.22 (0.89 - 1.68) |
| *TMCO1*** | rs4656461 | C | | | 0,32 | | 0,28 | | 0.1501 | 1.29 (0.91 - 1.83) |
| *TMCO1*** | rs1913845 | T | | | 0,12 | | 0,13 | | 0.5084 | 0.85 (0.51 - 1.39) |
| *TMCO1*** | rs12059327 | C | | | 0,15 | | 0,13 | | 0.4069 | 1.22 (0.76 - 1.95) |
| *TMCO1*** | rs6426940 | C | | | 0,40 | | 0,40 | | 0.6474 | 0.93 (0.67 - 1.29) |
| *TMCO1*** | rs7518099 | C | | | 0,08 | | 0,07 | | 0.3560 | 1.32 (0.73 - 2.36) |
| *TMCO1*** | rs2814471 | C | | | 0,17 | | 0,16 | | 0.7302 | 1.08 (0.7 - 1.67) |
| *COL8A2* | rs274750 | T | | | 0,45 | | 0,44 | | 0.8861 | 0.98 (0.72 - 1.32) |
| ***COL8A2*** | **rs6693322** | **G** | | | **0,11** | | **0,07** | | **0.0139** | **1.92 (1.14 - 3.24)** |
| *COL8A2* | rs274754 | A | | | 0,35 | | 0,35 | | 0.9099 | 0.98 (0.72 - 1.35) |
| *MYOC* | rs61730975 | T | | | 0,03 | | 0,03 | | 0.7059 | 0.84 (0.33 - 2.1) |
| *MYOC* | rs61745146 | T | | | 0,03 | | 0,02 | | 0.6215 | 1.27 (0.49 - 3.26) |
| *MYOC* | rs61730976 | A | | | 0,05 | | 0,03 | | 0.4166 | 1.43 (0.6 - 3.39) |
| *MYOC* | rs79255460 | A | | | 0,03 | | 0,04 | | 0.9238 | 0.96 (0.45 - 2.05) |
| *MYOC* | rs12076134 | G | | | 0,20 | | 0,20 | | 0.8841 | 0.97 (0.68 - 1.4) |
| *MYOC* | rs2032555 | G | | | 0,07 | | 0,06 | | 0.7202 | 1.12 (0.61 - 2.03) |
| *MYOC* | rs57824969 | A | | | 0,00 | | 0,01 | | 0.8166 | 0.79 (0.11 - 5.69) |
| *MYOC* | rs235869 | C | | | 0,30 | | 0,27 | | 0.5757 | 1.1 (0.78 - 1.56) |
| *MYOC* | rs16864720 | T | | | 0,14 | | 0,16 | | 0.5256 | 0.87 (0.57 - 1.33) |
| *MYOC* | rs235876 | C | | | 0,07 | | 0,06 | | 0.6987 | 1.12 (0.63 - 2.02) |
| *MYOC* | rs2236875 | T | | | 0,20 | | 0,18 | | 0.7228 | 1.07 (0.73 - 1.56) |
| ***MYOC*** | **rs235917** | **A** | | | **0,08** | | **0,04** | | **0.0140** | **2.32 (1.19 - 4.52)** |
| *MYOC* | rs7525674 | C | | | 0,04 | | 0,04 | | 0.8723 | 0.94 (0.43 - 2.03) |
| *MYOC* | MYOCnc1 | G | | | 0,02 | | 0,03 | | 0.7262 | 1.18 (0.46 - 3.05) |
| *MYOC* | rs61730977 | G | | | 0,04 | | 0,05 | | 0.7431 | 0.88 (0.43 - 1.84) |
| *MYOC* | rs2234926 | T | | | 0,00 | | 0,01 | | 0.2387 | 0.26 (0.03 - 2.42) |
| *MYOC* | rs12082573 | G | | | 0,04 | | 0,04 | | 0.7710 | 0.89 (0.42 - 1.9) |
| *MYOC* | rs2075648 | T | | | 0,01 | | 0,01 | | 0.8599 | 1.13 (0.28 - 4.5) |
| *CYP1B1* | rs9341261 | G | | | 0,03 | | 0,04 | | 0.4858 | 0.74 (0.33 - 1.71) |
| *CYP1B1* | rs162562 | T | | | 0,32 | | 0,28 | | 0.4268 | 1.15 (0.82 - 1.62) |
| *CYP1B1* | rs1056836 | G | | | 0,17 | | 0,20 | | 0.1656 | 0.76 (0.52 - 1.12) |
| *CYP1B1* | rs162561 | T | | | 0,20 | | 0,17 | | 0.2433 | 1.25 (0.86 - 1.83) |
| *CYP1B1* | rs9341252 | G | | | 0,05 | | 0,04 | | 0.2384 | 1.54 (0.75 - 3.15) |
| *C2p16* | rs2303298 | A | | | 0,10 | | 0,11 | | 0.6792 | 0.9 (0.56 - 1.46) |
| *C2p16* | rs10202118 | A | | | 0,25 | | 0,26 | | 0.4288 | 0.87 (0.62 - 1.23) |
| *C2p16* | rs11125375 | T | | | 0,29 | | 0,22 | | 0.0898 | 1.33 (0.96 - 1.84) |
| *C2p16* | rs10208467 | G | | | 0,22 | | 0,25 | | 0.1922 | 0.79 (0.56 - 1.13) |
| *C2p16* | rs1533428 | A | | | 0,39 | | 0,37 | | 0.2472 | 1.19 (0.89 - 1.59) |
| *C2p16* | rs12994401 | T | | | 0,03 | | 0,02 | | 0.5070 | 1.39 (0.53 - 3.67) |
| *C2p16* | rs11889995 | A | | | 0,07 | | 0,08 | | 0.4147 | 0.79 (0.45 - 1.39) |
| *WDR36* | rs7729832 | A | | | 0,12 | | 0,12 | | 0.6203 | 1.13 (0.7 - 1.82) |
| *WDR36* | rs17623144 | C | | | 0,00 | | 0,00 | | 0.9993 | N/A |
| *WDR36* | rs13357724 | T | | | 0,01 | | 0,00 | | 0.5743 | 1.68 (0.27 - 10.38) |
| *WDR36* | rs13153937 | A | | | 0,05 | | 0,05 | | 0.7557 | 0.9 (0.47 - 1.74) |
| *WDR36* | rs6859041 | G | | | 0,29 | | 0,28 | | 0.6694 | 1.08 (0.76 - 1.53) |
| *WDR36* | rs2416257 | A | | | 0,13 | | 0,12 | | 0.5016 | 1.17 (0.74 - 1.87) |
| *WDR36* | rs17132783 | G | | | 0,17 | | 0,15 | | 0.3759 | 1.2 (0.8 - 1.81) |
| *WDR36* | rs10038058 | A | | | 0,25 | | 0,25 | | 0.9562 | 1.01 (0.7 - 1.45) |
| *WDR36* | rs12515367 | G | | | 0,03 | | 0,03 | | 0.9144 | 0.95 (0.4 - 2.29) |
| *WDR36* | rs12521169 | A | | | 0,14 | | 0,14 | | 0.9849 | 1 (0.66 - 1.54) |
| *WDR36* | rs10051830 | G | | | 0,29 | | 0,27 | | 0.5630 | 1.11 (0.79 - 1.56) |
| *WDR36* | rs10491424 | G | | | 0,47 | | 0,48 | | 0.5645 | 0.91 (0.67 - 1.25) |
| *WDR36* | rs11956837 | G | | | 0,03 | | 0,04 | | 0.7587 | 0.88 (0.39 - 2) |
| *WDR36* | rs12522383 | T | | | 0,17 | | 0,18 | | 0.7403 | 0.94 (0.64 - 1.38) |
| *WDR36* | rs17624563 | C | | | 0,18 | | 0,17 | | 0.6659 | 1.09 (0.73 - 1.62) |
| *WDR36* | rs43203 | T | | | 0,06 | | 0,06 | | 0.6692 | 0.87 (0.45 - 1.66) |
| *WDR36* | rs11241098 | T | | | 0,14 | | 0,16 | | 0.7674 | 0.94 (0.62 - 1.43) |
| *WDR36* | rs7722241 | T | | | 0,43 | | 0,40 | | 0.3115 | 1.18 (0.86 - 1.62) |
| *WDR36* | rs10041326 | C | | | 0,07 | | 0,08 | | 0.6207 | 0.88 (0.53 - 1.46) |
| *WDR36* | rs4530809 | G | | | 0,23 | | 0,19 | | 0.2633 | 1.23 (0.86 - 1.77) |
| *WDR36* | rs17132810 | T | | | 0,14 | | 0,15 | | 0.8352 | 0.96 (0.63 - 1.46) |
| *WDR36* | rs1457113 | C | | | 0,08 | | 0,06 | | 0.2842 | 1.39 (0.76 - 2.51) |
| *COL1A2* | rs4729131 | A | | | 0,08 | | 0,08 | | 0.8553 | 0.95 (0.55 - 1.64) |
| *COL1A2* | rs3814967 | A | | | 0,36 | | 0,39 | | 0.3851 | 0.87 (0.64 - 1.19) |
| *COL1A2* | rs1800222 | A | | | 0,37 | | 0,34 | | 0.3090 | 1.18 (0.86 - 1.61) |
| *COL1A2* | rs411717 | C | | | 0,27 | | 0,26 | | 0.9903 | 1 (0.71 - 1.42) |
| *COL1A2* | rs406226 | C | | | 0,13 | | 0,12 | | 0.7073 | 1.09 (0.69 - 1.73) |
| *COL1A2* | rs3763466 | A | | | 0,14 | | 0,14 | | 0.8771 | 0.97 (0.63 - 1.48) |
| *COL1A2* | rs17166249 | G | | | 0,29 | | 0,24 | | 0.2165 | 1.24 (0.88 - 1.75) |
| *COL1A2* | rs42518 | C | | | 0,10 | | 0,07 | | 0.1273 | 1.52 (0.89 - 2.61) |
| *COL1A2* | rs2521206 | C | | | 0,30 | | 0,34 | | 0.2284 | 0.83 (0.6 - 1.13) |
| *COL1A2* | rs42521 | C | | | 0,38 | | 0,36 | | 0.4723 | 1.12 (0.82 - 1.54) |
| *COL1A2* | rs42522 | G | | | 0,23 | | 0,22 | | 0.8877 | 0.97 (0.68 - 1.4) |
| *COL1A2* | rs42524 | G | | | 0,07 | | 0,06 | | 0.8556 | 1.06 (0.56 - 2.01) |
| *COL1A2* | rs42526 | A | | | 0,44 | | 0,43 | | 0.4935 | 1.11 (0.83 - 1.49) |
| *COL1A2* | rs2521205 | A | | | 0,34 | | 0,36 | | 0.8254 | 0.97 (0.71 - 1.32) |
| *COL1A2* | rs3736638 | T | | | 0,25 | | 0,29 | | 0.1824 | 0.8 (0.57 - 1.11) |
| *COL1A2* | rs42527 | A | | | 0,15 | | 0,14 | | 0.7551 | 1.07 (0.69 - 1.67) |
| *COL1A2* | rs369982 | T | | | 0,19 | | 0,16 | | 0.2360 | 1.28 (0.85 - 1.93) |
| *COL1A2* | rs4266 | G | | | 0,21 | | 0,22 | | 0.5361 | 0.89 (0.62 - 1.29) |
| *COL1A2* | rs10235102 | T | | | 0,08 | | 0,08 | | 0.8507 | 0.95 (0.55 - 1.64) |
| *COL1A2* | rs441051 | T | | | 0,27 | | 0,24 | | 0.3187 | 1.2 (0.84 - 1.69) |
| *COL1A2* | rs2072071 | A | | | 0,21 | | 0,24 | | 0.2097 | 0.8 (0.56 - 1.13) |
| *COL1A2* | rs13240759 | C | | | 0,12 | | 0,13 | | 0.7772 | 0.94 (0.61 - 1.45) |
| *COL1A2* | rs400218 | C | | | 0,20 | | 0,18 | | 0.4779 | 1.15 (0.78 - 1.71) |
| *COL1A2* | rs10046552 | G | | | 0,19 | | 0,21 | | 0.4924 | 0.88 (0.62 - 1.26) |
| *COL1A2* | rs12668754 | A | | | 0,02 | | 0,01 | | 0.3337 | 1.8 (0.55 - 5.92) |
| *COL1A2* | rs11764718 | T | | | 0,06 | | 0,05 | | 0.8118 | 1.08 (0.56 - 2.09) |
| *COL1A2* | rs1062394 | A | | | 0,11 | | 0,14 | | 0.4147 | 0.83 (0.53 - 1.3) |
| *COL1A2* | rs1034620 | C | | | 0,08 | | 0,07 | | 0.9565 | 1.02 (0.58 - 1.78) |
| *COL1A2* | rs11982782 | T | | | 0,47 | | 0,46 | | 0.9948 | 1 (0.74 - 1.35) |
| *CAV1&2* | rs8940 | C | | | 0,19 | | 0,19 | | 0.6760 | 0.92 (0.63 - 1.35) |
| *CAV1&2* | rs10258482 | T | | | 0,44 | | 0,41 | | 0.7793 | 1.04 (0.78 - 1.4) |
| *CAV1&2* | rs6466578 | C | | | 0,16 | | 0,14 | | 0.5950 | 1.12 (0.74 - 1.69) |
| *CAV1&2* | rs3919515 | C | | | 0,37 | | 0,41 | | 0.4245 | 0.89 (0.66 - 1.19) |
| *CAV1&2* | rs926198 | A | | | 0,49 | | 0,47 | | 0.5587 | 1.09 (0.81 - 1.47) |
| *CAV1&2* | rs3779512 | G | | | 0,37 | | 0,36 | | 0.9033 | 0.98 (0.74 - 1.31) |
| *CAV1&2* | rs10262090 | C | | | 0,11 | | 0,13 | | 0.9950 | 1 (0.65 - 1.55) |
| *CAV1&2* | rs3807986 | T | | | 0,28 | | 0,25 | | 0.5506 | 1.11 (0.79 - 1.54) |
| *CAV1&2* | rs1476833 | A | | | 0,29 | | 0,28 | | 0.7442 | 0.95 (0.68 - 1.32) |
| *CAV1&2* | rs10270569 | T | | | 0,18 | | 0,17 | | 0.7278 | 1.07 (0.72 - 1.6) |
| *CAV1&2* | rs11773845 | T | | | 0,23 | | 0,22 | | 0.4273 | 1.15 (0.81 - 1.64) |
| *CAV1&2* | rs9886215 | C | | | 0,32 | | 0,36 | | 0.3465 | 0.86 (0.62 - 1.18) |
| *CAV1&2* | rs3807992 | A | | | 0,48 | | 0,47 | | 0.5124 | 1.11 (0.82 - 1.49) |
| *CAV1&2*** | rs1052990 | C | | | 0,45 | | 0,44 | | 0.9953 | 1 (0.73 - 1.37) |
| *CAV1&2*** | rs10227696 | A | | | 0,17 | | 0,16 | | 0.6370 | 1.11 (0.72 - 1.73) |
| *CAV1&2*** | rs917664 | T | | | 0,48 | | 0,46 | | 0.7561 | 1.05 (0.76 - 1.45) |
| *CAV1&2*** | rs3807989 | C | | | 0,24 | | 0,21 | | 0.4339 | 1.17 (0.79 - 1.72) |
| *CAV1&2*** | rs3779514 | A | | | 0,23 | | 0,26 | | 0.3364 | 0.83 (0.57 - 1.21) |
| *CAV1&2*** | rs3815412 | G | | | 0,46 | | 0,46 | | 0.8050 | 0.96 (0.68 - 1.34) |
| *CAV1&2*** | rs8713 | C | | | 0,33 | | 0,36 | | 0.7504 | 0.95 (0.68 - 1.32) |
| *CDKN2B* | rs3217992 | A | | | 0,06 | | 0,05 | | 0.6974 | 1.14 (0.59 - 2.18) |
| *CDKN2B* | rs1063192 | C | | | 0,00 | | 0,00 | | 0.4585 | 2.58 (0.21 - 31.41) |
| *CDKN2B* | rs3217989 | C | | | 0,32 | | 0,29 | | 0.4686 | 1.13 (0.82 - 1.55) |
| *CDKN2B* | rs974336 | A | | | 0,23 | | 0,30 | | 0.0862 | 0.74 (0.52 - 1.05) |
| *CDKN2B* | rs3217980 | A | | | 0,08 | | 0,11 | | 0.1702 | 0.7 (0.42 - 1.17) |
| *CDKN2B* | rs2069422 | G | | | 0,11 | | 0,12 | | 0.7951 | 0.94 (0.58 - 1.52) |
| *CDKN2B-AS1*** | rs7049105 | A | | | 0,18 | | 0,20 | | 0.7862 | 0.94 (0.63 - 1.42) |
| *CDKN2B-AS1*** | rs2151280 | A | | | 0,22 | | 0,21 | | 0.6001 | 1.11 (0.75 - 1.66) |
| *CDKN2B-AS1*** | rs7851706 | T | | | 0,16 | | 0,14 | | 0.4332 | 1.2 (0.76 - 1.89) |
| *CDKN2B-AS1*** | rs10120688 | A | | | 0,38 | | 0,39 | | 0.9766 | 1.01 (0.72 - 1.39) |
| *CDKN2B-AS1*** | rs16905597 | A | | | 0,07 | | 0,10 | | 0.1849 | 0.67 (0.37 - 1.21) |
| *CDKN2B-AS1*** | rs16905599 | T | | | 0,26 | | 0,26 | | 0.9161 | 0.98 (0.69 - 1.4) |
| *CDKN2B-AS1*** | rs16923583 | A | | | 0,18 | | 0,20 | | 0.6743 | 0.92 (0.62 - 1.36) |
| *CDKN2B-AS1*** | rs1547705 | C | | | 0,22 | | 0,24 | | 0.5286 | 0.88 (0.6 - 1.3) |
| *CDKN2B-AS1*** | rs1537370 | C | | | 0,35 | | 0,31 | | 0.3542 | 1.19 (0.82 - 1.72) |
| *CDKN2B-AS1*** | rs10965235 | C | | | 0,47 | | 0,47 | | 0.9136 | 0.98 (0.7 - 1.37) |
| *CDKN2B-AS1*** | rs4990722 | T | | | 0,14 | | 0,13 | | 0.9064 | 1.03 (0.63 - 1.67) |
| *CDKN2B-AS1*** | rs17761446 | G | | | 0,04 | | 0,04 | | 0.7201 | 1.16 (0.52 - 2.56) |
| *CDKN2B-AS1*** | rs1333049 | C | | | 0,24 | | 0,24 | | 0.4936 | 0.88 (0.6 - 1.28) |
| *CDKN2B-AS1*** | rs1333050 | T | | | 0,12 | | 0,12 | | 0.7991 | 1.07 (0.66 - 1.73) |
| *CDKN2B-AS1*** | rs10811658 | G | | | 0,47 | | 0,48 | | 0.3099 | 0.85 (0.61 - 1.17) |
| *CDKN2B-AS1*** | rs12347779 | G | | | 0,02 | | 0,03 | | 0.5713 | 0.76 (0.29 - 1.97) |
| *CDKN2B-AS1*** | rs10965245 | A | | | 0,24 | | 0,26 | | 0.8392 | 0.96 (0.67 - 1.39) |
| *CDKN2B-AS1*** | rs2383208 | G | | | 0,24 | | 0,24 | | 0.2564 | 1.24 (0.85 - 1.8) |
| *COL5A1* | rs1536478 | A | | | 0,29 | | 0,29 | | 0.5892 | 0.92 (0.67 - 1.26) |
| *COL5A1* | rs3118516 | A | | | 0,19 | | 0,15 | | 0.4068 | 1.18 (0.8 - 1.75) |
| *COL5A1* | rs1536482 | T | | | 0,46 | | 0,43 | | 0.3961 | 1.14 (0.84 - 1.54) |
| *COL5A1* | rs7044529 | T | | | 0,39 | | 0,41 | | 0.1946 | 0.82 (0.61 - 1.11) |
| *ATOH7* | rs6480320 | A | | | 0,29 | | 0,27 | | 0.6329 | 1.08 (0.78 - 1.5) |
| *ATOH7* | rs7916697 | G | | | 0,20 | | 0,23 | | 0.4840 | 0.88 (0.61 - 1.27) |
| *ATOH7* | rs2289804 | C | | | 0,50 | | 0,49 | | 0.6546 | 1.07 (0.8 - 1.43) |
| *ATOH7* | rs1900004 | C | | | 0,24 | | 0,24 | | 0.6538 | 1.09 (0.76 - 1.55) |
| *ATOH7* | rs41340944 | G | | | 0,43 | | 0,46 | | 0.6165 | 0.93 (0.69 - 1.25) |
| *ATOH7* | rs1900020 | G | | | 0,49 | | 0,46 | | 0.5999 | 1.08 (0.81 - 1.45) |
| *SIX1&6* | rs12436579 | T | | | 0,47 | | 0,51 | | 0.2588 | 0.84 (0.63 - 1.13) |
| *SIX1&6* | rs17097585 | T | | | 0,09 | | 0,08 | | 0.5682 | 1.17 (0.69 - 1.98) |
| *SIX1&6* | rs8006274 | T | | | 0,27 | | 0,25 | | 0.5323 | 1.11 (0.8 - 1.55) |
| *SIX1&6* | rs1007152 | G | | | 0,27 | | 0,25 | | 0.5546 | 1.11 (0.79 - 1.55) |
| *SIX1&6* | rs7152532 | A | | | 0,03 | | 0,02 | | 0.6471 | 1.25 (0.48 - 3.22) |
| *SIX1&6* | rs11849906 | G | | | 0,05 | | 0,03 | | 0.0590 | 2.22 (0.97 - 5.08) |
| *SIX1&6* | rs1955698 | A | | | 0,43 | | 0,47 | | 0.2605 | 0.84 (0.63 - 1.14) |
| *SIX1&6* | rs10150234 | G | | | 0,43 | | 0,46 | | 0.4369 | 0.89 (0.66 - 1.2) |
| *SIX1&6* | rs11158289 | T | | | 0,40 | | 0,42 | | 0.5633 | 0.91 (0.67 - 1.24) |
| *SIX1&6* | rs10142401 | G | | | 0,32 | | 0,33 | | 0.6600 | 0.93 (0.68 - 1.28) |
| *SIX1&6* | rs10483726 | C | | | 0,48 | | 0,50 | | 0.3998 | 0.88 (0.66 - 1.18) |
| *SIX1&6* | rs8020353 | T | | | 0,26 | | 0,26 | | 0.9032 | 1.02 (0.73 - 1.43) |
| *SIX1&6* | rs10483727 | G | | | 0,01 | | 0,01 | | 0.9034 | 0.92 (0.25 - 3.43) |
| *SIX1&6* | rs2057135 | C | | | 0,44 | | 0,43 | | 0.7842 | 1.04 (0.77 - 1.4) |
| *SIX1&6* | rs7156317 | C | | | 0,50 | | 0,48 | | 0.4651 | 1.12 (0.83 - 1.49) |
| *SIX1&6* | rs7146104 | A | | | 0,07 | | 0,06 | | 0.8204 | 1.07 (0.59 - 1.95) |
| *SIX1&6* | rs4901995 | C | | | 0,15 | | 0,14 | | 0.7598 | 1.07 (0.71 - 1.6) |
| *SIX1&6* | rs8007935 | A | | | 0,23 | | 0,19 | | 0.2231 | 1.25 (0.87 - 1.8) |
| *SIX1&6* | rs11628064 | T | | | 0,09 | | 0,10 | | 0.5763 | 0.87 (0.53 - 1.42) |
| *SIX1&6*** | rs2350890 | C | | | 0,48 | | 0,50 | | 0.3904 | 0.86 (0.62 - 1.21) |
| *SIX1&6*** | rs4901977 | G | | | 0,48 | | 0,48 | | 0.8347 | 0.97 (0.71 - 1.33) |
| *SIX1&6*** | rs8012339 | C | | | 0,24 | | 0,24 | | 0.9856 | 1 (0.69 - 1.44) |
| *SIX1&6*** | rs1266416 | C | | | 0,36 | | 0,38 | | 0.3657 | 0.86 (0.62 - 1.2) |
| *SIX1&6*** | rs3759688 | A | | | 0,21 | | 0,19 | | 0.4002 | 1.18 (0.8 - 1.73) |
| *SIX1&6*** | rs10148202 | A | | | 0,14 | | 0,12 | | 0.6664 | 1.1 (0.7 - 1.73) |
| *ZNF469* | rs12447690 | T | | | 0,14 | | 0,15 | | 0.9925 | 1 (0.65 - 1.54) |
| *ZNF469* | rs7500824 | C | | | 0,16 | | 0,17 | | 0.7033 | 0.92 (0.62 - 1.38) |
| *ZNF469* | rs4352077 | A | | | 0,06 | | 0,05 | | 0.8781 | 1.05 (0.54 - 2.04) |
| *ZNF469* | rs7501402 | T | | | 0,25 | | 0,24 | | 0.7324 | 1.07 (0.74 - 1.53) |
| *ZNF469* | rs6540223 | C | | | 0,33 | | 0,29 | | 0.3505 | 1.17 (0.85 - 1.61) |
| *ZNF469* | rs12448211 | A | | | 0,33 | | 0,38 | | 0.0856 | 0.75 (0.55 - 1.04) |
| *ZNF469* | rs9938149 | C | | | 0,44 | | 0,38 | | 0.1099 | 1.29 (0.94 - 1.75) |
| ***ZNF469*** | **rs9925231** | **A** | | | **0,50** | | **0,43** | | **0.0225** | **1.43 (1.05 - 1.93)** |
| *ZNF469* | rs11644804 | T | | | 0,25 | | 0,26 | | 0.8545 | 0.97 (0.68 - 1.37) |
| *COL1A1* | rs2696297 | C | | | 0,16 | | 0,19 | | 0.3061 | 0.82 (0.55 - 1.2) |
| ***COL1A1*** | **rs16948744** | **G** | | | **0,37** | | **0,44** | | **0.0143** | **0.68 (0.5 - 0.93)** |
| *COL1A1* | rs2696296 | T | | | 0,42 | | 0,46 | | 0.1203 | 0.79 (0.59 - 1.06) |
| *COL1A1* | rs36107109 | T | | | 0,09 | | 0,10 | | 0.6764 | 0.9 (0.55 - 1.48) |
| *COL1A1* | rs2586476 | T | | | 0,31 | | 0,33 | | 0.5000 | 0.9 (0.65 - 1.23) |
| *COL1A1* | rs12945599 | T | | | 0,08 | | 0,07 | | 0.5178 | 1.2 (0.69 - 2.07) |
| *COL1A1* | rs2696270 | C | | | 0,43 | | 0,39 | | 0.2893 | 1.18 (0.87 - 1.6) |
| *COL1A1* | rs1061947 | T | | | 0,15 | | 0,18 | | 0.2260 | 0.79 (0.53 - 1.16) |
| *COL1A1* | rs1061237 | C | | | 0,48 | | 0,47 | | 0.8192 | 1.04 (0.77 - 1.4) |
| *COL1A1* | rs2277632 | C | | | 0,46 | | 0,40 | | 0.0779 | 1.31 (0.97 - 1.78) |
| *COL1A1* | rs2696245 | G | | | 0,07 | | 0,07 | | 0.6582 | 1.14 (0.64 - 2.02) |
| *COL1A1* | rs2586488 | T | | | 0,50 | | 0,48 | | 0.2793 | 1.18 (0.87 - 1.59) |
| *COL1A1* | rs2075559 | G | | | 0,39 | | 0,45 | | 0.0963 | 0.77 (0.56 - 1.05) |
| *COL1A1* | rs2857396 | C | | | 0,09 | | 0,09 | | 0.6419 | 1.13 (0.69 - 1.85) |
| *COL1A1* | rs2696247 | C | | | 0,15 | | 0,14 | | 0.6651 | 1.1 (0.72 - 1.67) |
| *COL1A1* | rs2586494 | T | | | 0,20 | | 0,17 | | 0.2234 | 1.28 (0.86 - 1.89) |
| *COL1A1* | rs16948765 | C | | | 0,06 | | 0,08 | | 0.3067 | 0.75 (0.43 - 1.31) |
| *COL1A1* | rs2586498 | G | | | 0,36 | | 0,33 | | 0.4894 | 1.12 (0.82 - 1.52) |
| The genotyping was performed using the BeadXpress platform on 215 POAG participants and 214 controls except where ** denotes TaqMan genotyping that was successful in 179 POAG participants and 187 controls | | | | | | | | | | |
| MA, minor allele; MAF, minor allele frequency; OR (95% CI), Odds ratio (95% confidence intervals) | | | | | | | | | |  |
| Bold indicates significance at the level of p<0.05 | | |  |  | |  | |  | |  |

Supplementary Table 2

| Supplementary Table 2: The genetic association of SNPs in *COL1A1, COL1A2, COL5A1* and *COL8A2* and the region of *ZNF469* with CCT using linear regression modeling with the justification of age, gender and POAG diagnosis (POAG or control) | | | | | | | |
| --- | --- | --- | --- | --- | --- | --- | --- |
| **Genomic region** | **SNP** | **Regression Coefficient** | **P** | **Genomic region** | **SNP** | **Regression Coefficient** | **P** |
| *COL8A2* | rs274750 | -1,914 | 0,5794 | *COL1A2* | rs1034620 | -1,359 | 0,8331 |
| *COL8A2* | rs6693322 | 4,516 | 0,4405 | *COL1A2* | rs11982782 | 0,6685 | 0,8457 |
| *COL8A2* | rs274754 | -2,826 | 0,4416 | *COL5A1* | rs1536478 | 0,4485 | 0,9016 |
| *CYP1B1* | rs9341261 | -4,41 | 0,6433 | *COL5A1* | rs3118516 | 0,6232 | 0,8871 |
| *CYP1B1* | rs10916 | 0,9181 | 0,7871 | *COL5A1* | rs1536482 | -1,017 | 0,7632 |
| *CYP1B1* | rs162562 | 1,028 | 0,7952 | *COL5A1* | rs7044529 | -4,315 | 0,2088 |
| *CYP1B1* | rs1056836 | -1,825 | 0,6692 | *ZNF469* | rs12447690 | 7,986 | 0,1036 |
| *CYP1B1* | rs162561 | 1,169 | 0,7865 | ***ZNF469*** | **rs7500824** | **8,851** | **0,04724** |
| *CYP1B1* | rs9341252 | 2,475 | 0,754 | *ZNF469* | rs4352077 | 3,607 | 0,6371 |
| *COL1A2* | rs4729131 | 4,534 | 0,4577 | *ZNF469* | rs7501402 | 6,276 | 0,1272 |
| *COL1A2* | rs3814967 | -6,064 | 0,08668 | *ZNF469* | rs6540223 | -3,108 | 0,3966 |
| *COL1A2* | rs1800222 | 2,074 | 0,5597 | *ZNF469* | rs12448211 | 2,917 | 0,419 |
| *COL1A2* | rs411717 | 1,556 | 0,6965 | *ZNF469* | rs9938149 | -5,024 | 0,1444 |
| *COL1A2* | rs406226 | 4,683 | 0,3662 | *ZNF469* | rs9925231 | 1,656 | 0,6305 |
| *COL1A2* | rs3763466 | -4,571 | 0,3491 | *ZNF469* | rs11644804 | 5,176 | 0,1942 |
| *COL1A2* | rs17166249 | 3,398 | 0,3767 | *COL1A1* | rs2696297 | -0,2401 | 0,9569 |
| *COL1A2* | rs42518 | 8,482 | 0,1622 | *COL1A1* | rs16948744 | -1,459 | 0,6716 |
| **COL1A2** | **rs2521206** | **-7,284** | **0,04486** | *COL1A1* | rs2696296 | -3,489 | 0,2946 |
| *COL1A2* | rs42521 | 0,4202 | 0,9075 | *COL1A1* | rs36107109 | -3,184 | 0,5681 |
| *COL1A2* | rs42522 | 6,044 | 0,141 | *COL1A1* | rs2586476 | -0,6172 | 0,8647 |
| *COL1A2* | rs42524 | 12,54 | 0,08127 | *COL1A1* | rs12945599 | 6,386 | 0,2997 |
| *COL1A2* | rs42526 | 3,007 | 0,3818 | *COL1A1* | rs2586481 | -2,938 | 0,5314 |
| *COL1A2* | rs2521205 | 3,453 | 0,3323 | *COL1A1* | rs2696270 | 0,8907 | 0,7946 |
| *COL1A2* | rs3736638 | -6,549 | 0,08815 | *COL1A1* | rs1061947 | -3,471 | 0,4317 |
| *COL1A2* | rs42527 | -4,509 | 0,3679 | *COL1A1* | rs1061237 | -2,917 | 0,4024 |
| *COL1A2* | rs369982 | 1,855 | 0,685 | *COL1A1* | rs2277632 | 3,294 | 0,3387 |
| *COL1A2* | rs4266 | 1,685 | 0,6855 | *COL1A1* | rs2696245 | 4,266 | 0,5306 |
| *COL1A2* | rs10235102 | 9,504 | 0,1505 | *COL1A1* | rs2586488 | -3,247 | 0,343 |
| *COL1A2* | rs441051 | -1,224 | 0,7582 | *COL1A1* | rs2075559 | -4,545 | 0,1975 |
| *COL1A2* | rs2072071 | -5,864 | 0,1429 | *COL1A1* | rs2857396 | 4,286 | 0,4611 |
| *COL1A2* | rs13240759 | 0,5091 | 0,917 | *COL1A1* | rs2696247 | -3,04 | 0,5371 |
| *COL1A2* | rs400218 | 3,664 | 0,4006 | *COL1A1* | rs2586494 | 1,69 | 0,7072 |
| *COL1A2* | rs10046552 | 4,819 | 0,2415 | *COL1A1* | rs2075554 | -8,016 | 0,2395 |
| *COL1A2* | rs12668754 | -9,311 | 0,4907 |  |  |  |  |
| *COL1A2* | rs11764718 | 12,18 | 0,1104 |  |  |  |  |
| *COL1A2* | rs1062394 | 0,3577 | 0,947 |  |  |  |  |
| Bold signifies p<0.05 | | | | | | | |

Supplementary Table 3

| Supplementary Table 3: The genetic association of SNPs in the regions of *CDKN2B, SIX1/SIX6,* and *ATOH7* and vertical cup-to-disc ratio using linear regression modeling with the justification of age, gender and POAG diagnosis (POAG or control) | | | | | | | |
| --- | --- | --- | --- | --- | --- | --- | --- |
| **Genomic region** | **SNP** | **Regression Coefficient** | **P** | **Genomic region** | **SNP** | **Regression Coefficient** | **P** |
| *CDKN2B* | rs3217992 | 0,08293 | 0,4266 | *SIX1&6* | rs7152532 | 0,02713 | 0,8615 |
| *CDKN2B* | rs1063192 | 0,1969 | 0,6327 | *SIX1&6* | rs11849906 | 0,1181 | 0,3647 |
| *CDKN2B* | rs3217989 | -0,03474 | 0,5077 | *SIX1&6* | rs1955698 | -0,0403 | 0,4061 |
| *CDKN2B* | rs974336 | -0,03342 | 0,5621 | *SIX1&6* | rs10150234 | -0,03633 | 0,4512 |
| *CDKN2B* | rs3217980 | -0,1255 | 0,1349 | *SIX1&6* | rs11158289 | -0,03715 | 0,4579 |
| *CDKN2B* | rs2069422 | 0,0326 | 0,6823 | *SIX1&6* | rs10142401 | 0,002195 | 0,9664 |
| *ATOH7* | rs6480320 | 0,06548 | 0,2161 | *SIX1&6* | rs10483726 | -0,03358 | 0,4814 |
| *ATOH7* | rs7916697 | 0,04702 | 0,4233 | *SIX1&6* | rs8020353 | 0,06251 | 0,2535 |
| *ATOH7* | rs2289804 | 0,04486 | 0,3461 | *SIX1&6* | rs10483727 | -0,07122 | 0,7308 |
| *ATOH7* | rs1900004 | -0,05056 | 0,3783 | *SIX1&6* | rs2057135 | 0,04277 | 0,3731 |
| *ATOH7* | rs41340944 | -0,01393 | 0,7711 | *SIX1&6* | rs7156317 | 0,06055 | 0,2002 |
| *ATOH7* | rs1900020 | 0,004247 | 0,9299 | *SIX1&6* | rs7146104 | 0,0565 | 0,5578 |
| *SIX1&6* | rs12436579 | -0,03292 | 0,4991 | *SIX1&6* | rs4901995 | -0,006866 | 0,9172 |
| *SIX1&6* | rs17097585 | -0,04864 | 0,5699 | *SIX1&6* | rs8007935 | 0,06874 | 0,2418 |
| *SIX1&6* | rs8006274 | 0,078 | 0,1475 | *SIX1&6* | rs11628064 | -0,08193 | 0,3096 |
| *SIX1&6* | rs1007152 | -0,02757 | 0,6184 |  |  |  |  |
